# Association of *TP53* gene codon 72 polymorphism with endometriosis risk in Isfahan

**Published:** 2013-06

**Authors:** Mehdi Nikbakht Dastjerdi, Roshanak Aboutorabi, Bahram Eslami Farsani

**Affiliations:** 1 *Department of Anatomical Sciences and Molecular Biology, Faculty of Medicine, Isfahan University of Medical Sciences, Isfahan, Iran.*

**Keywords:** *Polymorphism*, *Genotype*, *P53 gene*, *Endometriosis*

## Abstract

**Background: **Endometriosis is a female health disorder that occurs when cells from the lining of the uterus grow in other areas of the body. The cause of endometriosis is unknown.

**Objective:** The purpose of this study was to investigate *TP53* gene codon 72 polymorphism in women with endometriosis and compared it with healthy samples in Isfahan.

**Materials and Methods: **We undertook a case-control study to examine the possible association of the *TP53* gene codon 72 polymorphism with the risk of endometriosis in Isfahan. Ninety whole blood specimens from normal people as controls and ninety endometriosis specimens were analyzed. p53 codon 72 genotypes were identified using allele-specific polymerase chain reaction.

**Results:** Frequency of genotype Arg/Arg (Arginine/Arginine) in the samples of endometriosis was 28.9% and in healthy samples 42.2%. Frequency of genotype Pro/Pro (Proline/Proline) in the samples of endometriosis was 15.6% and in healthy ones. Frequency of heterozygote's Arg/Pro was 55.6% in endometriosis samples and 54.45% in healthy ones 3.3%. By comparing statistical genotype Pro/Pro with two other genotypes in both groups there was a statistical meaningful difference between control group and endometriosis group. [p=0.009, CI=95%, OR=5.34 (1047-19.29)].

**Conclusion:** Recent research shows that genotype Pro/Pro codon72 exon4 *TP53* gene may be one predisposing genetic factor for endometriosis in Isfahan.

## Introduction

Endometriosis is one of the most common gynecological problems in women. It has the same features of malignant tumors and its cause is unknown. Almost 10% of women suffer from this disease and it is seen in 18% of women during the reproductive years (1, 2). This ratio reaches to nearly 30-40% in infertile women (3). Endometriosis has been occurred by growing endometrial tissues outside the uterus and causes the abnormalities like painful sexual intercourse, painful menstruation, pelvic pains and infertility (4-7).

For explaining the cause of the disease, theory of retrograde menstruation is the most widely accepted theory. It suggests that some endometrial debris exit the uterus through the fallopian tubes and aggregate with each other on the peritoneal surface where it can proceed to invade the tissue as endometriosis (8). This event occurs during a woman's menstrual flow. Some of genetic deficits may have the role in making disease (9). It is determined that the chromosome 17 may be impaired in this disease, because *TP53* gene as the most important tumor suppressor gene that is located on short arm of chromosome 17 (10-12). 

The genetic instability of cancer cells is a common event which is caused by aneuploidy and gene mutation. Chromosome 17 aneuploidy had occurred during the development of cancer. The product of the *TP53* gene, has named p53 protein. This protein participates in cell cycle regulation, apoptosis, DNA repair, and angiogenesis and is mutated in more than 50% of all cancers (13, 14). The *TP53 *gene consists of 11 exons and 10 introns and has a high frequency of mutations and polymorphisms (15). 

Because endometriosis is believed to be a multifactorial illness and its frequency show a discrepancy, there is a necessity for persisted study to recognize polymorphisms that are related with the incidence of endometriosis and its unreliable medical stages. Even though it is indistinct which *TP53* gene polymorphism is accountable for changes in p53 protein action, a number of researches have revealed that polymorphisms in intron 3 and exon 4 are concerned in the growth of a variety of diseases (16). 

It have showed that structural alterations in the p53 protein can outcome from a single amino acid exchange that causes improper protein action and defeat of role because of an incapability to cooperate with further transcription factors for transcription. These changes can cause carcinogenesis and cancer development (16). It is determined that codon 72 exon 4 *TP53* gene has epidemic polymorphism that may produce two alleles. One allele is Arginine (Arg) with the sequence of CGC and other one is Proline (Pro) with sequence of CCC. According to the possibility of existing these alleles, it is possible to create three different genotypes such as Arg/Arg, Pro/Arg, Pro/Pro (16-24). 

The codon 72 polymorphism is placed in a SH3-binding domain site, which is necessitated for development repression and apoptosis arbitrated by p53 but not for cell cycle stop (16). The proteins p53Arg72 and p53Pro72 have dissimilar biochemical and biological possessions for example dissimilarity in the binding to parts of the transcriptional apparatus and dissimilarity in the initiation of transcription (17). The p53Arg72 protein persuades apoptosis quicker and represses alteration more competently than the p53Pro72 protein (17, 18). 

Because of efficient divergences between the two polymorphic alternatives of *TP53*, genotype at codon 72 could change susceptibility to endometriosis growth. The function of codon 72 polymorphism of *TP53 *gene had been noticed in the patients with endometrosis in several populations even though the outcomes are still contentious (25-32). It had been shown that the codon 72 polymorphism in different geographic and ethnic populations have different effects on disease (19). However, the function of the polymorphism concerning endometriosis risk in the Isfahanian population has not been accounted. 

This study discovers a probable relationship between endometriosis and this polymorphism in a minor population choosed from Isfahan metropolis.

## Materials and methods

This cross-sectional study was conducted between February 2010 and august 2011 in Shahid Beheshti, Mehregan and Alzahra hospitals of Isfahan and Department of Anatomy and Molecular Biology of Isfahan University of Medical Sciences. Samples were divided into two groups: 1) endometriosis (n=90); and 2) nonendometriosis group (n=90). The non-endometriosis statuses as control group were confirmed during the cesarean section or diagnostic laparoscopy. 

In endometriosis group, women with surgically and histologically diagnosed endometriosis were included, the criterion to enter the study was confirmation which obtained from pathologist and gynecologist in diagnosis of endometriosis, stage II to stage IV. Stage I or minimal (mild endometriosis) excluded from this study since this stage could be observed in asymptomatic women and maybe considered as normal physiological process. Again the cases with structural abnormality in genital system, hypertensive and diabetic women were excluded from our study. Informed consent was obtained from each woman after a brief and clarified explanation was given to them on why their samples are being obtained for this study. 

The Ethics Committee of Isfahan University of Medical Sciences approved this study. After gathering the samples, DNA was extracted by standard methods as described previously (33). In the next stage, amplification of exon 4 of *TP53 *was performed using Alle-specific PCR with the following primers: 5'-TTGCCGTCCCAAGCAATGGATGA-3' and 5'-TCTGGGAAGGGACAGAAGATGAC-3' (Pairs of specific primers for Arginine) or 5′GCCAGAGGCTGCTCCCCC-3' and 5′-CGTGCAAGTCACAGACTT-3' (Pairs of specific primers for Proline) (18). Among 100-300 nanograms DNA was utilized as template in a 25 µL PCR reaction combination including 1.5 µmol MgCl_2_, 1 U Taq polymerase (Sinagen, Iran) and 2 µmol either of the primer pairs. 

PCR cycling circumstances were carried out with an early denaturation step for 3 min at 94^o^C, followed by 35 cycles of 30 s at 94^o^C, 30 s at 60^o^C (for Arg) or 54^o^C (for Pro) and 30 s at 72^o^C. A finishing extension step was executed at 72^o^C for 5 min. The PCR reaction was done individually for each of the two polymorphic variants. The amplified outcomes were subjected to electrophoresis on 1% agarose gel in 1×TBE buffer and visualized on a transilluminator by means of ethidium bromide. We used a PCR 50 bp Ladder in exact 100 bp spaced recombinant repeats (Sinagen, Iran).

**Statistical analysis**

Obtained information via software SPSS version 20 have been analyzed. For comparing the frequency distribution of three different genotype of codon 72 in endometriosis samples with that of control ones, Chi-Square Test has been used. P-value less than 0.05% is considered meaningful.

## Results

**Figure 1:**
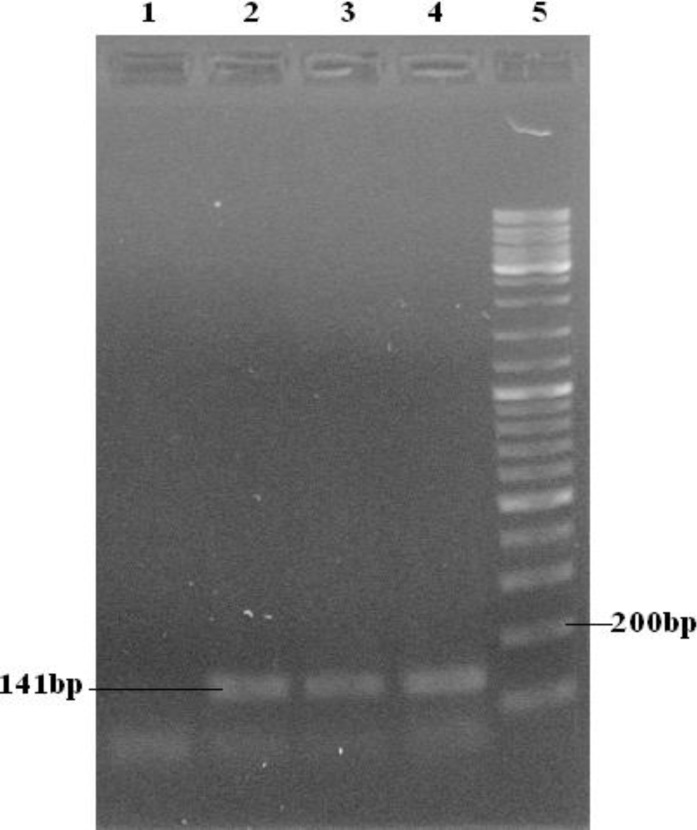
Agarose gel electrophoresis for Arginine allele: samples 2-4 have the band (141bp). Lane 5 is ladder and lane 1 is negative control. Present bands in the end of lanes are primer dimmer

**Figure 2:**
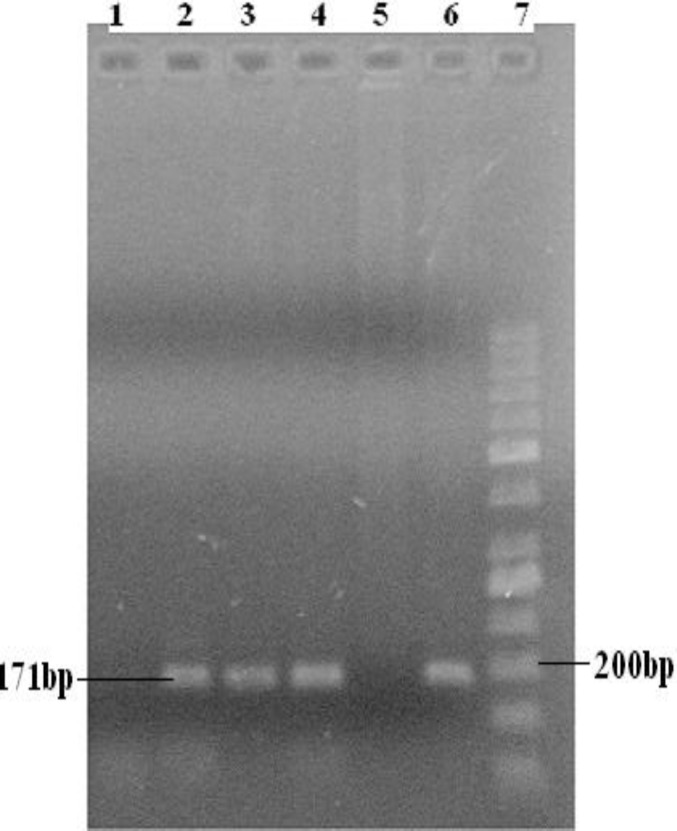
Agarose gel electrophoresis for Proline allele (171bp): samples 2-4 and 6 have the band and sample 5 has not the band. Lane 7 is ladder and lane1 is negative control. Present bands in the end of lanes are primer dimmer

The ages of samples were between 23-50 years old. To analyze the codon 72 polymorphism, we used a PCR-based assay that specifically, amplify either *TP53* Pro or *TP53* Arg allele and give a PCR product by using specific primers for Arg allele and/or Pro allele respectively. Consequential PCR products were either 141bp (Figure 1) for Arg allele or 177bp (Figure 2) for Pro allele.

*TP53* codon 72 polymorphism was successfully detected by allele -specific PCR in all cases and controls. The distribution of the three different genotypes of codon 72 in exon 4 of *TP53* in our cases and controls is shown in table I. In control samples, the genotype distribution for p53 polymorphism showed 42.2%, 54.4% and 3.3% for the Arg/Arg, Arg/Pro and Pro/Pro genotypes, respectively. In the endometriosis group 28.9% of the cases were Arg/Arg, 55.6% were Arg/Pro and 15.6% were Pro/Pro (Table I). A significant difference between cases and controls was found for the Pro/Pro genotype compared with (grouped) Arg/Pro and Arg/Arg genotypes (OR=5.34 (1.47-19.29), p=0.009).

**Table I:** Genotypic frequency distribution in control and endometriosis group

**Genotype**	**Endometriosis**	**Control group**
	**n (%)**	**N (%)**
Arg/ Arg	26 (28.9)	38 (42.2)
Pro/ Pro	14 (15.6)	3 (3.3)
Arg/ Pro	50 (55.6)	49 (54.4)

## Discussion

In this study, we found a difference in frequency distribution of genotype between control and endometriosis. Frequency of genotype Arg/Pro in patient group is 55.6% in comparison with 54.4% in healthy group. Frequency of genotype Arg/Arg in patient group is 28.9% in comparison with 42.2% in healthy group. Frequency of genotype Pro/Pro in patients group is 15.6% in comparison with 3.3% in healthy group. Therefore, results of this study showed that Pro/Pro genotype can be considered as probable risk of endometriosis in Isfahan [p=0.009, CI: 95%, OR=5.34 (1047-19.29)].

This finding is compatible with the result representing the situation of Chinese population by the Change *et al* and also Lattuada’s group in the Milan city (26, 32). Chang and his colleagues reported that there is a relationship between Pro allele and endometriosis among Chinese population. The proportions of Arg homozygotes, heterozygote, and Pro homozygotes in the endometriosis and nonendometriosis groups in their study were 10.2%, 66.9%, 22.9% and 30.7%, 50%, and 19.3%, respectively. The study has been conducted on a group of Milanese people in Italy, Lattuada and his colleagues found highest frequency of Proline allele in more critical kinds (26). 

The respective proportions of arginine homozygotes, heterozygotes and proline homozygotes in their study were 55.6, 39.7 and 4.6% in the group with endometriosis and 59.9, 30.9 and 9.2% in the control group. Also Ammendola *et al* studied the interaction between *TP53* and *PTPN22* and found that the Arg genotype showed a protective effect against endometriosis in carriers of the T allele of *PTPN22* (27). In the study of Brazilian women, Ribeiro Júnior and his colleagues found a significant association between the heterozygous and homozygous proline genotypes and intense pain in the patients (28).

Ghasemi *et al* reported an increased frequency of the Pro allele in Iranian women with endometrial cancer and observed that in infertile women with endometriosis, Pro allele is very common (29). The respective proportions of arginine homozygotes, heterozygotes and proline homozygotes in their study were 7, 50 and 43% in the group with endometriosis and 12, 66 and 22% in the control group. These studies showed an association between the Pro/Pro and heterozygote genotypes of the *TP53 *gene and endometriosis. Homozygous genotype Arg/Arg in codon 72 has low aptitude to suffer endometriosis, as homozygous or heterozygous allele of Pro has higher aptitude to suffer endometriosis.

Endometriosis occurs in roughly 6-10% of women. This ratio is 30-40% in infertile. A number of histopathological studies point that endometriosis might be the neoplastic procedure leading to endometrial ovarian and clear cell carcinoma, most expected resulting from accelerated propagation of endometrial glands and stroma exterior of the uterine cavity (34, 35). 

The *TP53* tumor suppressor gene holds a high incidence of missense mutations that are chiefly established in the central DNA-binding domain that can change gene regulation and protein levels and influence cell development, DNA repair, and apoptosis and endorse carcinogenesis (16). Molecular mutations like polymorphism of *TP53* relate to growing and developing this disease. Natural role of protein P53 is to protect genome against received damages and leads to repair genome and /or apoptosis (15). 

Recently, a number of studies have investigated genetic polymorphisms as a possible factor contributing to the development of endometriosis. The relationship between *TP53* gene polymorphisms and endometriosis is contentious; some researches account a negative relationship between endometriosis risk and the *TP53* codon 72 polymorphism. These researches propose that this negative connection is affected by ethnical variations (36). 

Other researches propose an enhanced risk of endometriosis in patients with the *TP53* codon 72 polymorphism (26-32). Our results suggest that the frequencies of *TP53* codon 72 Pro/Pro and Arg/Pro genotypes are significantly different between control women and endometriosis patients. So, these data indicate that the proline allele of codon 72 *TP53* polymorphism might be a good marker for endometriosis patients in the Isfahanian population. The proline allele of codon 72 polymorphism might influence mRNA splicing and influence gene expression and DNA-protein communications. 

This might produce changes in the endometrial tissue, and when paired with oxidative stress, may participate a central position in the inflammatory response of endometriosis, proposed that the increase of pro-oxidant things as the heme group and iron present in retrograde menstruation and ovarian hemorrhage can effect in toxicity and probably play a part to endometriosis and the succeeding malignant alteration (34). Unlike mentioned repots, recent results of Omori *et al* have not found the relation between endometriosis and polymorphism of codon pro72 (25). In their report, the percentages of Arg homozygotes/ heterozygotes/ Pro homozygotes in endometriosis and control groups were 35.2/48.6%/16.2% and 39.4/41.7/18.9%, respectively. 

This inconsistency may be because of racial difference. In another study, metaanalyses discovered that persons with the Pro-carriers had increased risk of endometriosis (OR=2.595, 95%; CI:1.005-6.702, p=0.049) in Asian, but not in Caucasian (OR=1.005, 95%; CI:0.755-1.337, p=0.972) (31). So, the differences in ethnicity may be one major cause for the disagreement a variety of speculations attempt to clarify the mechanism of endometriosis. However, further studies with greater sample size is necessary, along with regard to other factors that can weaken the immune system such as smoking, diet and susceptible to certain diseases.
